# Work for the Wounded of the Allied Armies

**Published:** 1914-11-21

**Authors:** 


### HOSPITALS AND THE WAR.

(From our Special Correspondents.)

### Work for the Wounded of the Allied Armies.

### NELSON HOSPITAL, WIMBLEDON.

In order to show that the smaller London hospitals are doing their share in the reception and treatment of wounded soldiers, such an instance may be taken as that of the Nelson Hospital for Wimbledon. The first draft of wounded sent to this hospital arrived on November 5, and consisted of four men: one Scot (Argyll and Sutherland Highlanders), one Irishman (Leinsters), one Englishman (Coldstream Guards), and one Belgian. Others are expected shortly, and the fact that the smaller hospitals have been asked to assist in the work is the best proof, if further evidence were required, how large a burden is being undertaken.

### BATH.

Eighteen of the Belgian wounded who have been at the Royal United Hospital left recently for London, whence they will soon return to the Front. The men were driven to the Great Western Railway Station in motor-cars and received a good "send off" from those who had assembled on the platform, among them being Dr. Leckie, acting house surgeon; Mr. J. M. Sheppard, secretary, and several members of the nursing staff.

### BRIGHTON: 2nd EASTERN GENERAL HOSPITAL ENLARGED.

When first mobilised, this Territorial hospital was arranged to contain 520 beds in two buildings, the Brighton and Hove Grammar School and the Stanford Road Council School, with a staff of three administrative officers, eighteen medical officers, 119 non-commissioned officers and men, and about 120 sisters and nurses. Owing to the great demand for accommodation, not only for wounded men from the Expeditionary Force but also for the Territorials and the men of Kitchener's Army who become sick or who require operation (*e.g.*, for hernia, for varicocele, for varicose veins, for deformities, etc.), the War Office have decided to increase the number of beds to 1,000, making it one of the largest military hospitals in England. For this purpose it has been necessary not only to acquire additional buildings, but also to increase the staff and *personnel* of the hospital. The extra accommodation is made up as follows. The number of beds in the present buildings has been increased to 609, in the two new buildings taken over—the Howard Convalescent Home and the St. Mark's Church School—there are respectively 73 and 160 beds, making a total of 842. Another building close by is to be acquired shortly to bring the available accommodation up to 1,000 beds. The new buildings are situated in Kemp Town, at the extreme east of Brighton, in quite open country, and are well suited for the purpose for which they have been adapted. They contain all the necessary ward accommodation, an operating room, a dispensary, rooms for the medical officers and the administrative staff, etc., and make up a really well-equipped hospital. The nursing staff has been increased by the addition of about forty nurses, and those engaged at the Kemp Town Hospital are being housed and boarded at a neighbouring convent. The administrative staff has not so far been increased, but promotions have been made as follows: Major James A. Rooth has received the rank of lieutenant-colonel, and Lieutenant Walker has been promoted to captain.

Up to the present only eighteen of the thirty medical officers available on mobilisation have been called up at a time, but this number is now being added to. Up to date six convoys of wounded from the English Expeditionary Force, totalling 800 men, and two convoys of wounded Belgians, numbering 181, have been admitted. This includes some who have been taken to the Royal Sussex County Hospital and to the French Convalescent Home. From the home forces (Kitchener's Army and the Territorials) 988 admissions have been made.

In the 981 cases of wounded men from the Front there have only been nine deaths, the cause of which is as follows: Two from tetanus, five from bullet wounds, one from typhoid, and one from meningitis. About two hundred operations have been performed in the only theatre so far used, the main one at the Grammar School, where a second theatre is being provided.

### SUNDERLAND: THE SECOND CONVOY.

The second convoy of wounded soldiers from oversea to be sent to Sunderland arrived on November 3. The ambulance train left Southampton with 130 cases, one was detrained at Oxford (a case of severe hæmorrhage), and fifty were taken off at York, the remainder coming on to Sunderland, where they arrived about 5.30 A.M. There were fifty "cot" cases. The men were conveyed to the Royal Infirmary by members of the Voluntary Aid Detachment, under the direction of Mr. Claude Palmer, Deputy Commissioner, and Mr. E. Seymour Wood, inspector of stores, who had at their disposal about a dozen ambulance vans from the neighbouring collieries, and a large number of private motor-cars. The majority of the first convoy have been discharged convalescent. Use is now being made of the various temporary hospitals in the town for the treatment of convalescent soldiers from the base hospital at Newcastle-on-Tyne, and the Red Cross Society is rendering valuable assistance in this work.

### COVENTRY.

Further batches of wounded soldiers have been recently received at the Coventry and Warwickshire Hospital. The earlier arrivals came, *via* Southampton, straight from the trenches at the Front. In reply to an intimation on the previous day asking the hospital if they could take in a number of wounded men, a wire was dispatched stating that the hospital would be willing to admit forty wounded. The beds in the Cox-Spencer and Chater Wards were immediately prepared. The special ambulance train, containing forty wounded, arrived at the Coventry Station about 7.15. The removal of the soldiers from the train to the hospital was efficiently carried out by the nurses and bearers of the 26th Division of the Red Cross Society. Hot Bovril and coffee were given. The Daimler Motor Company kindly provided Red Cross ambulance waggons for the "cot" cases, and motor-cars were supplied by the Division of the Red Cross Society to take the sitting-up cases. On their passage through the city to the hospital a great deal of subdued enthusiasm was displayed. Each man who was able took a bath, and as the majority of them had not had their clothes off since the commencement of the war, their happiness can be imagined.

Every member of the hospital staff worked extremely hard. Another sixteen wounded men have been since admitted to the hospital from the 1st Southern Military Hospital, Birmingham; eleven were Belgians.

The hospital is providing further accommodation for the reception of wounded soldiers. The house committee having decided to fit up the reserve premises at the back of the hospital as a temporary ward, in another few days the ward will be complete and furnished to admit another forty men. Towards this a handsome donation has been made by Mr. Alfred Herbert, on behalf of his firm, of £1,000.

